# U87MG Decoded: The Genomic Sequence of a Cytogenetically Aberrant Human Cancer Cell Line

**DOI:** 10.1371/journal.pgen.1000832

**Published:** 2010-01-29

**Authors:** Michael James Clark, Nils Homer, Brian D. O'Connor, Zugen Chen, Ascia Eskin, Hane Lee, Barry Merriman, Stanley F. Nelson

**Affiliations:** 1 Department of Human Genetics, University of California Los Angeles, Los Angeles, California, United States of America; 2 Department of Computer Science, University of California Los Angeles, Los Angeles, California, United States of America; University of Washington, United States of America

## Abstract

U87MG is a commonly studied grade IV glioma cell line that has been analyzed in at least 1,700 publications over four decades. In order to comprehensively characterize the genome of this cell line and to serve as a model of broad cancer genome sequencing, we have generated greater than 30× genomic sequence coverage using a novel 50-base mate paired strategy with a 1.4kb mean insert library. A total of 1,014,984,286 mate-end and 120,691,623 single-end two-base encoded reads were generated from five slides. All data were aligned using a custom designed tool called BFAST, allowing optimal color space read alignment and accurate identification of DNA variants. The aligned sequence reads and mate-pair information identified 35 interchromosomal translocation events, 1,315 structural variations (>100 bp), 191,743 small (<21 bp) insertions and deletions (indels), and 2,384,470 single nucleotide variations (SNVs). Among these observations, the known homozygous mutation in *PTEN* was robustly identified, and genes involved in cell adhesion were overrepresented in the mutated gene list. Data were compared to 219,187 heterozygous single nucleotide polymorphisms assayed by Illumina 1M Duo genotyping array to assess accuracy: 93.83% of all SNPs were reliably detected at filtering thresholds that yield greater than 99.99% sequence accuracy. Protein coding sequences were disrupted predominantly in this cancer cell line due to small indels, large deletions, and translocations. In total, 512 genes were homozygously mutated, including 154 by SNVs, 178 by small indels, 145 by large microdeletions, and 35 by interchromosomal translocations to reveal a highly mutated cell line genome. Of the small homozygously mutated variants, 8 SNVs and 99 indels were novel events not present in dbSNP. These data demonstrate that routine generation of broad cancer genome sequence is possible outside of genome centers. The sequence analysis of U87MG provides an unparalleled level of mutational resolution compared to any cell line to date.

## Author Summary

Glioblastoma has a particularly dismal prognosis with median survival time of less than fifteen months. Here, we describe the broad genome sequencing of U87MG, a commonly used and thus well-studied glioblastoma cell line. One of the major features of the U87MG genome is the large number of chromosomal abnormalities, which can be typical of cancer cell lines and primary cancers. The systematic, thorough, and accurate mutational analysis of the U87MG genome comprehensively identifies different classes of genetic mutations including single-nucleotide variations (SNVs), insertions/deletions (indels), and translocations. We found 2,384,470 SNVs, 191,743 small indels, and 1,314 large structural variations. Known gene models were used to predict the effect of these mutations on protein-coding sequence. Mutational analysis revealed 512 genes homozygously mutated, including 154 by SNVs, 178 by small indels, 145 by large microdeletions, and up to 35 by interchromosomal translocations. The major mutational mechanisms in this brain cancer cell line are small indels and large structural variations. The genomic landscape of U87MG is revealed to be much more complex than previously thought based on lower resolution techniques. This mutational analysis serves as a resource for past and future studies on U87MG, informing them with a thorough description of its mutational state.

## Introduction

Grade IV glioma, also called glioblastoma multiforme (GBM), is the most common primary malignant brain tumor with about 16,000 new diagnoses each year in the United States. While the number of cases is relatively small, comprising only 1.35% of primary malignant cancers in the US [1], GBMs have a one-year survival rate of only 29.6%, making it one of the most deadly types of cancer [2]. Recent clinical studies demonstrate improved survival with a combination of radiation and Temozolomide chemotherapy, but median survival time for GBM patients who receive therapy is only 15 months [3]. Due to its highly aggressive nature and poor therapeutic options, understanding the genetic etiology of GBM is of great interest and therefore, GBM has been selected as one of the three initial cancer types to be thoroughly studied in the TCGA program [4].

To that end, numerous cell line models of GBM have been established and used in vast numbers of studies over the years. It is well recognized that cell line models of human disorders, especially cancers, are an important resource. While these cell lines are the basis of substantial biological insight, experiments are currently performed in the absence of genome-wide mutational status as no cell line that models a human disease has yet had its genome fully sequenced. Here, we have sequenced the genome of U87MG, a long established cell line derived from a human grade IV glioma used in over 1,700 publications [5]. A wide range of biological information is known about this cell line. The U87MG cell line is known to have a highly aberrant genomic structure based on karyotyping, SKY [6], and FISH [7]. However, these methods neither provide the resolution required to visualize the precise breakpoint of a translocation event, nor are they generally capable of identifying genomic microdeletions (deletions on the order of a megabase or less in size) in a whole genome survey of structural variation. SNP genotyping microarrays can be used to detect regions of structural variation in the forms of loss of heterozygosity (LOH) and copy number (CN) based on probe intensity, but do not reveal chromosomal joins. To assess the genomic stability of U87MG, the genome was genotyped by Illumina Human 1M-Duo BeadChip microarray. In spite of being cultured independently for several years, the regions of LOH and the CN state of our U87MG genome matched exactly with data retrieved from the Sanger COSMIC database for U87MG [8], which had been assayed on an Affymetrix Genome-Wide Human SNP Array 6.0. This suggests that although U87MG bears a large number of large-scale chromosomal aberrations, it has been relatively stable for years and is not rapidly changing. This suggests that prior work on U87MG may be reinterpreted based on the whole genome sequence data presented here.

The first draft of the consensus sequence of the human genome was reported in 2001 [9],[10]. The first individual human diploid sequence was sequenced using capillary-based Sanger sequencing [11]. Since then, a few additional diploid human genomes have been published utilizing a variety of massively parallel sequencing techniques to sequence human genomes to varying degrees of coverage, variant discovery, and quality typically costing well over $200,000 and several machine months of operation [12]–[16]. For the sequencing of U87MG, we utilized ABI SOLiD technology, which uses a ligation-based assay with two-base color-encoded oligonucleotides that has been demonstrated to allow highly accurate single nucleotide variant (SNV) and insertion/deletion (indel) detection [17]. Additionally, long mate-paired genomic libraries with a mean insert size of 1–2kb allowed higher clone coverage of the genome, which improved our ability to identify genomic structural variations such as interchromosomal translocations and large deletions. While longer insert sizes would improve resolution of some structural variants, during genomic shearing the highest density of large fragments occurs at 1.5kb, allowing a sufficiently complex library to be generated from only 10 micrograms of genomic DNA while still being well powered to identify structural variations. Here, we demonstrate that aligning the two-base color-encoding data with BFAST software and decoding during alignment allows for highly sensitive detection of indels, which have in the past been difficult to detect by short read massively parallel sequencing.

For cancer sequencing, it is important to assess not only SNVs, but indels, structural variations and translocations, and it is preferable to extract this information from a common assay platform. A major characteristic of the U87MG cell line that differentiates it from the samples used in other whole genome sequencing projects published thus far is its highly aberrant genomic structure. Due to its heavily rearranged state, we thoroughly and accurately assessed each of these major classes of mutations and demonstrated that small indels, large microdeletions and interchromosomal translocations are actually the major categories of mutations that affect known genes in this cancer cell line. These analyses provide a model for other genome sequencing projects outside major genome centers of how to both thoroughly sequence and assess the mutational state of whole genomes.

## Results

### Data Production

From ten micrograms of input genomic DNA, we performed two and a half full sequencing runs on the ABI SOLiD Sequencing System, for a total of five full slides of data [17]. Utilizing the ABI long mate-pair protocol, we produced 1,014,984,286 raw 50bp mate-paired reads (101.5Gb). In some cases the bead was recognized by the imaging software for only one read, thereby producing an additional 120,691,623 single end reads (6.0Gb). In aggregate, we generated a total of 107.5Gb of raw data (Table 1).

**Table 1:** Genome sequencing summary.

**Sequencing Libraries**	1
**SOLiD Runs (Slides)**	2.5 (5)
**Strategy**	2×50
**Mate-paired reads passing quality filter (total bases)**	1,014,984,286 (101.5Gb)
**Single-end reads passing quality filter (total bases)**	120,691,623 (6.0Gb)
**Mate-paired reads uniquely aligned by BFAST (bases)**	390,064,184 (39.06Gb)
**Unpaired reads uniquely aligned by BFAST (bases)**	266,635,829 (13.33Gb)
**Single-end reads uniquely aligned by BFAST (bases)**	62,336,824 (3.12Gb)
**Total bases uniquely aligned by BFAST**	55.51Gb

We also performed an exon capture approach designed to sequence the exons of 5,253 genes (10.7Mb) annotated in the Wellcome Trust Sanger Institute Catalogue of Somatic Mutations in Cancer (COSMIC) V38 [8], Cancer Gene Census, Cancer Genome Project Planned Studies and The Cancer Genome Atlas (TCGA) [4] GBM gene list using a custom-created Agilent array. This approach used the Illumina GAII sequencing system [18] to sequence captured DNA fragments using a paired end sequencing protocol. This resulted in 9,948,782 raw 76bp paired end reads (1.51Gb), and a mean base coverage of 29.5×. These reads were used to calculate concordance rates with the larger whole genome sequence dataset.

The Blat-like Fast Accurate Search Tool (BFAST) [19] version 0.5.3 was used to align 107.5Gb of raw color space reads to the color space conversion of the human genome assembly hg18 from University of California, Santa Cruz (http://hgdownload.cse.ucsc.edu/goldenPath/hg18/bigZips/, based on the March 2006 NCBI build 36.1). Duplicate reads, typically from the same initial PCR fragment during genomic library construction, were inevitable and accounted for 16.4% of the total aligned data. These were removed using the alignment filtering utility in the DNAA package (http://dnaa.sourceforge.net). A total of 390,604,184 paired end reads (39.06Gb), 266,635,829 (13.33Gb) unpaired reads, and 62,336,824 (3.12Gb) single end reads were successfully mapped to a unique location in the reference genome with high confidence for a total of 55.51Gb of aligned sequence (Table 1). For the exon capture dataset, we uniquely aligned 8,142,874 paired end reads (1.2Gb) and 1,097,000 (83Mb) unpaired reads for a total of 1.32Gb of raw aligned sequence (Table 2). Using the ABI SOLiD reads, we identified small insertions and deletions (indels), single nucleotide variants (SNVs), and structural variants such as large-scale microdeletions and translocation events. The exon capture Solexa reads were used to validate SNVs identified in the SOLiD sequencing.

**Table 2:** Exon capture sequencing summary.

**Sequencing Libraries**	1
**Illumina Runs (Lanes)**	1/8 (1)
**Strategy**	2×76
**Number of bases targeted**	10752923
**Mate-paired reads passing quality filter (total bases)**	9,948,782 (1.51Gb)
**Mate-paired reads uniquely aligned by BFAST (bases)**	8,142,874 (1.2Gb)
**Unpaired reads uniquely aligned by BFAST (bases)**	1,097,000 (83Mb)
**Total bases uniquely aligned by BFAST**	1.32Gb
**Total targeted bases sequenced**	317,017,503
**Mean coverage within targeted bases**	29.5×

**Figure 1:**
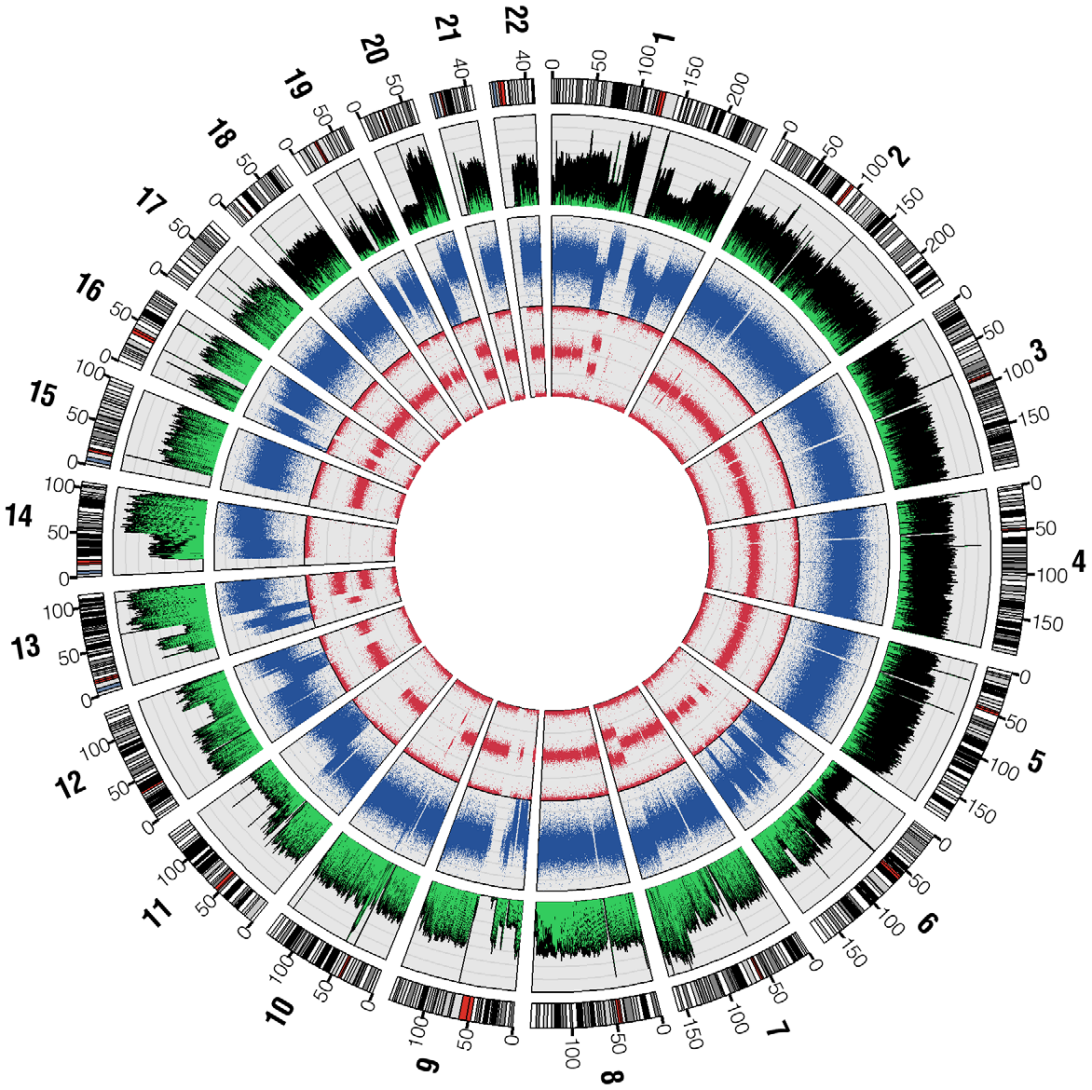
SNP chip and base sequence coverage compared. Circos [36] was used to plot base sequence coverage from ABI sequencing as a histogram (the outermost plot, green and black) alongside dot plots of the LogR-ratio (middle plot, blue) and B-allele frequency (innermost plot, red) from the Illumina Human 1M Duo BeadChip microarray. LogR-ratio represents copy number (CN) and B-allele frequency represents loss of heterozygosity (LOH).

The overall pattern of base sequence coverage from the shotgun reads changes across the genome, and as expected is highly concordant with the copy number state as determined by Illumina 1M Duo and Affymetrix 6.0 SNP analysis (Figure 1). Regions of two normal copies, such as chromosome 3, showed even base sequence coverage across their entire length (12.4 reads/base, excluding centromeric and telomeric regions which are not represented accurately in hg18). Meanwhile, regions with one-copy state according to the SNP chip, such as the distal q-arm of chr11 and the distal p-arm of chr6, show about half the base sequence coverage (7.2 reads/base) as a predicted two-copy region. Likewise, predicted three-copy state regions, such as the distal q-arm of chr13, show about 1.5 times the base sequence coverage of a predicted two-copy region. A complete deletion spanning the region on chromosome 9 that includes the CDKN2A gene is also seen in both the SNP chip and ABI SOLiD base sequence coverage. These data show at a very large scale that sequence placement is generally correct and supports the copy number state calls from the array based data.

### Variant Discovery

Single nucleotide variants (SNVs) and small insertions and deletions ranging from 1 to 20 bases (indels) were identified from the alignment data using the MAQ consensus model [20] as implemented in the SAMtools software suite [21]. SAMtools produced variant calls, zygosity predictions, and a Phred-scaled probability that the consensus is identical to the reference. To improve the reliability of our variant calls, variants were required to have a Phred score of at least 10 and further needed to be observed greater than or equal to 4 but less than 60 times and at least once on each strand.

In total, we identified 2,384,470 SNVs meeting our filtering criteria. Of these, 2,140,848 (89.8%) were identified as exact matches to entries in dbSNP129 [22]. Exact matches had both the variant and observed alleles in the dbSNP entry, allowing for the discovery of novel alleles at known SNP locations. In total, 243,622 SNVs (10.2%) were identified as novel events not previously recorded in dbSNP 129. This rate of novel variant discovery is consistent with other whole human normal genome sequences of European ancestry relative to dbSNP [12]. These SNVs were further characterized based on zygosity predictions from the MAQ consensus model, separating SNVs into homozygous or heterozygous categories (Table 3). The observed diversity value for SNVs (θ_SNV_, number of heterozygous SNVs/number of base pairs) across autosomal chromosomes was 4.4×10^−4^, which is generally consistent with the normal human genome variation rate.

**Table 3:** SNV filtering and quantification.

SNV Classification	Total	In dbSNP 129	Not in dbSNP 129
**Variants meeting filter criteria**	2,384,470	2,140,848	243,622
**Synonymous or not coding region**	2,375,812	2,133,226	242,586
**Coding region & non-synonymous**	8,658	7,622	1,036
**Splice site mutations**	151	132	19
**Heterozygous splice site mutations**	62	47	15
**Homozygous splice site mutations**	89	85	4
**Premature stop**	134	93	41
**Heterozygous premature stop**	82	48	34
**Homozygous premature stop**	52	45	7
**Non-synonymous**	8,538	7,518	1,020
**Heterozygous non-synonymous**	4,005	3,134	871
**Homozygous non-synonymous**	4,533	4,384	149

For small (<21bp) insertions and deletions, 191,743 events were detected with 116,964 not previously documented in dbSNP 129. The same criteria as used for SNVs was used for determining if an indel was novel and they were further classified as homozygous or heterozygous using the SAMtools variant caller (Table 4). The observed diversity value (θ_indel_, number of heterozygous indels/number of base pairs) across autosomal chromosomes was 0.38×10^−4^.

**Table 4:** Indel filtering and quantification.

Indel Classification	Total	In dbSNP 129	Not in dbSNP 129
**Variants meeting filter criteria**	191,743	74,779	116,964
**Synonymous or not coding region**	191,359	74,643	116,716
**Coding region & non-synonymous**	384	136	248
**Splice site mutations**	84	34	50
**Heterozygous splice site mutations**	20	7	13
**Homozygous splice site mutations**	64	27	37
**Heterozygous premature stop**	91	15	76
**Homozygous premature stop**	94	40	54
**Heterozygous non-synonymous**	168	45	123
**Homozygous non-synonymous**	193	86	107
**Heterozygous frameshift**	141	33	108
**Homozygous frameshift**	179	80	99
**Heterozygous in-frame indels**	26	11	15
**Homozygous in-frame indels**	14	5	9

A subset of 38 variants meeting genome-wide filtering criteria, including a 20-base deletion, was tested by PCR and Sanger sequencing with 34 being validated. In summary, 85.2% of SNVs (23/27), and 100% of small insertions (3/3), deletions (4/4), translocations (3/3) and microdeletions (1/1) were validated in this manner (Table S1). While this is a small sample, it demonstrates an overall low false positive rate.

### Indel Size Distribution

**Figure 2:**
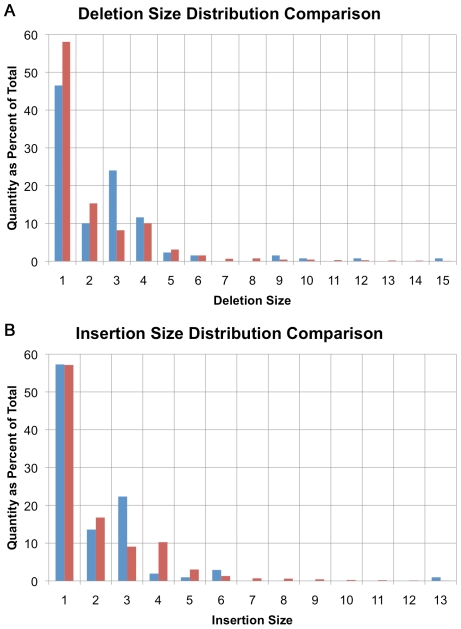
Small insertion/deletion size distribution. (A) Distribution of small deletion sizes as a percent of total, comparing amino-acid encoding deletions (blue) with non-coding deletions (red). (B) Distribution of small insertion sizes as a percent of total, comparing amino-acid encoding insertions (blue) with non-coding insertions (red).

The size distribution of indels identified in U87MG is generally consistent with previous studies on coding and non-coding indel sizes in non-cancer samples [23]–[25]. Small deletion sizes ranged from 1 to 20 bases in size and their distribution approximates a power law distribution in concordance with previous findings [23] (Figure 2A). There is a small deviation from the power law distribution with an excess of 4-base indels in U87MG's non-coding regions (Figure 2A, red bars) [11],[26].

A similar trend is seen with insertions in non-coding sequence with the maximum observed insertion size of 17 bases (Figure 2B, red bars). The maximum insertion size observed is less than the maximum deletion size because it is easier to align longer deletions than it is to align insertions. Some small insertions and deletions are likely to be larger than the upper limit of 17 and 20 bases actually observed, but the 50-base read length limits the power to align such reads directly.

In coding regions, there is a bias towards events that are multiples of 3-bases in length that maintain the reading frame despite variant alleles, suggesting that many of these are polymorphisms (Figure 2A-deletions, Figure 2B-insertions, blue bars). In non-coding regions, only 10.8% of indels are a multiple of 3 bases in size, while in coding regions, 27.0% are 3, 6, 9, 12 or 15 bases in size. This trend is expected based on past observations of non-cancer samples [11],[26].

### Nucleotide Substitution Frequencies

**Figure 3:**
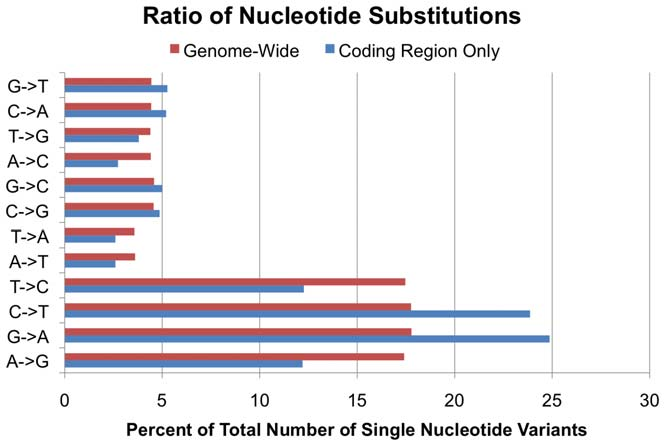
Base substitution frequencies. Ratio of specific nucleotide substitutions as a percent of total single nucleotide variants, comparing SNVs in coding regions (blue) to SNVs genome-wide (red).

Observed SNV base substitution patterns were consistent with common mutational phenomena in both coding sequences and genome wide. As expected, the predominant nucleotide substitution seen in SNVs is a transition, changing purine for purine (A<->G) or pyrimidine for pyrimidine (C<->T). Previous studies have observed that two out of every three SNPs are transitions as opposed to transversions [27], and we observed that 67.4% of our SNVs were transitions, while 32.6% were transversions, a 2.07∶1 ratio. (Figure 3) However, in coding regions, there appears to be an increase in C->T/G->A transitions and a decrease in T->C/A->G transitions, whereas genome-wide these transitions were approximately equivalent.

### Estimation of Genomic Coverage

To assess the coverage depth of the U87MG genome sequence, we followed Ley at al. [13] and required detection of both alleles at most positions in the genome. We utilized the Illumina 1M-Duo BeadChip to find reliably sequenced positions in the genome with an understanding that this may lead to bias towards more unique regions of the genome. In order to best use the SNP genotyping array data, we included only those regions that are diploid based on normal frequency of heterozygous calls and copy number assessment. This effectively permitted us to use the heterozygous calls for assessing accuracy of the short read data for variant calling (Figure 1). Only SNPs both observed to be heterozygous and that the Illumina genotyping chip called ‘high quality’ were used, which provided a total of 219,187 high quality heterozygous SNPs for comparison. 99.71% of these were sequenced at least once. After applying variant detection filtering criteria (see Materials and Methods) and assessing concordance between the sequence calls and genotyping array calls, 93.71% of the genome was sequenced at sufficient depth to call both alleles of the diploid genome. This is roughly equivalent to the likelihood of sufficient sampling of the whole genome when repeats and segmental duplications are excluded.

Notably, a variant allele was observed at every position called heterozygous by SNP chip, while a reference allele was observed at 201,414 (97.94%) positions. In other words, the SNV detection algorithm uniformly miscalled the homozygous variant allele. Filtering for quality causes a bias toward identifying SNVs at sites that have higher coverage. That said, after SNV quality filtering, diploid coverage of the cytogenetically normal portions of the genome was 10.85× for each allele, which is clearly adequate for calling over 90% of the base variant positions on each allele at high accuracy.

Because the positions of the genome included on SNP arrays is not a random sampling of the genome, we also assessed mapping coverage genome-wide. Of all bases in the haploid genome, 78.9% of the whole reference genome was covered by at least one reliably placed read. Of that portion of the genome, 91.9% of all bases were effectively sequenced based on passing variant calling filters (Phred>10, >4× coverage, <60× coverage). Thus, a total of 72.5% of the whole genome was sequenced, including repeats and duplicated regions, which is typical of short sequence shotgun approaches.

### Exon Capture Cross-Validation of Sequence Variants

10.9Mb of genomic sequence was targeted consisting of the amino acid encoding exons of 5,235 genes and were sequenced to a mean coverage of 30× using the Illumina GAII sequencer. Given the larger variability of coverage from the capture data, only a subset of these bases (8.5Mb) was evaluable to determine the false positive variant detection rate from the complete genomic sequence data. This region contained 1,621 SNPs present in dbSNP129. Within the 8.5Mb of common and well-covered sequence in the genomic sequence data and the capture sequence, there were 1,780 SNVs called from the genomic sequence. The same non-reference allele was concordantly observed at 1,631 positions within the capture data. At 149 positions, the non-reference allele was not observed in the capture data, but the reference allele was detected. However, the mean coverage at these 149 positions was significantly lower than that of the other 1,631 positions (p = 0.0003), suggesting that the non-reference allele was not adequately covered and is under called in the capture data. Moreover, of the 1,621 dbSNPs in the region, the capture adequately covered only 1,515. In these data there was a bias for the pull down data to under observe the non-reference allele (Figure S1). The 106 dbSNP positions detected in the ABI whole genome sequence dataset were observed to all call the reported alternate allele from dbSNP. In theory, if these were errors, then non-reference base calls should be randomly distributed to the three alternate base calls. Thus, no discrepancies are reliably identified within the dbSNP overlap when a variant was called in the ABI genomic sequence data.

There were a total of 100 novel SNVs detected in the ABI genomic sequence dataset that were also very well evaluated in the Illumina pull down data with at least 20 high quality Illumina reads, such that the ABI sequence could be well validated. Of these, 2 of the 100 discovered variants in the genomic sequence dataset were not observed in the Illumina pull down sequencing dataset. Thus, of the entire 8.5mb interval there are 2 unconfirmed variants for an estimated false positive error rate of about 3×10^−7^ for the whole interval. Alternatively viewed, there were 100 novel SNVs, with a 2% error rate in those novel positions. Thus, the de novo false discovery rate may be as high as 2%. Extrapolating to the whole set of 243,622 novel SNVs, we expect up to 4,872 false positives SNVs. These observations are roughly concordant with a sampling of 37 novel SNVs (not in dbSNP) in the whole genome set selected for testing by Sanger sequencing. Of these, 34 out of 37 (92%) were validated.

### Individual Genome Comparison

**Figure 4:**
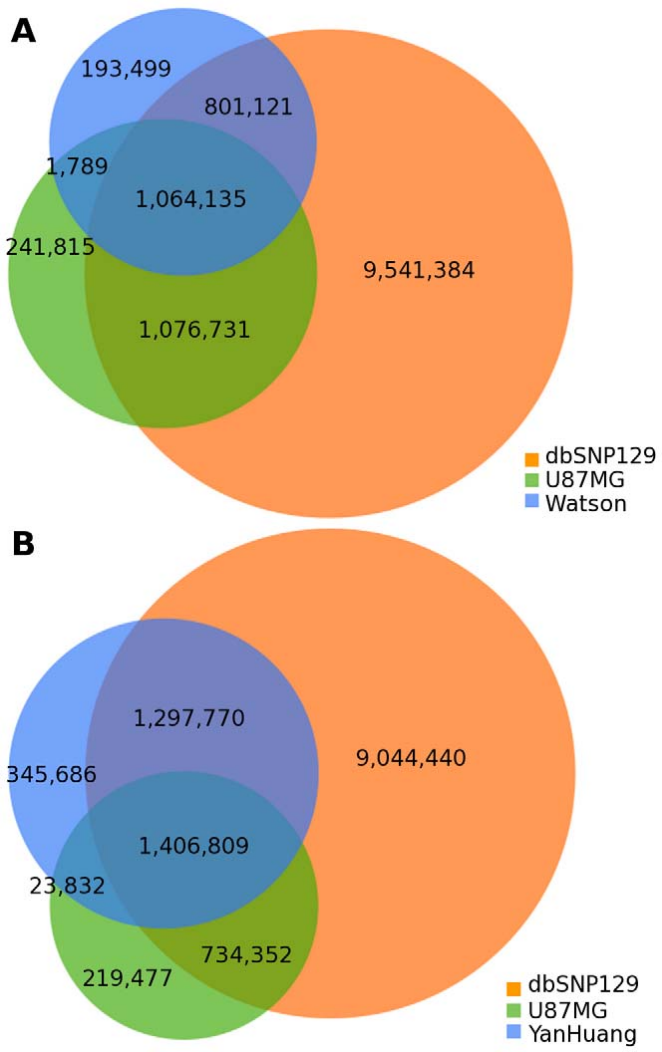
Individual genome comparison. (A) Venn diagram showing overlap in SNVs among the U87 genome, the Watson genome, and dbSNP 129. (B) Venn diagram showing overlap in SNVs among the U87 genome, the YanHuang genome, and dbSNP 129.

There are now several publicly available complete genomes sequenced on next generation platforms. We compared the SNVs discovered in U87MG to two of these published genomes: the James D. Watson genome [12] and the first Asian genome (YanHuang) [14]. Further, we simultaneously compared each of these to dbSNP version 129 [22]. Compared with dbSNP, 10.2% of U87MG SNVs, 9.5% of Watson SNVs, and 12.0% of YanHuang SNVs were not present within dbSNP (Figure 4). As U87MG was derived from a patient of Caucasian ancestry, which is confirmed by genotyping, it is unsurprising to see a higher overlap with dbSNP for U87MG than for YanHuang. Between the three genomes themselves, 44.7% of U87MG SNVs overlapped with Watson SNVs while 60.0% of SNVs were in common with YanHuang SNVs. Only 8.5% of dbSNP SNVs were shared between Watson and U87MG, while 11.3% of them were shared between YanHuang and U87MG. Thus, there is not a substantially higher amount of SNVs in the U87MG cancer genome relative to normal genomes.

### Structural Variation Identification

**Figure 5:**
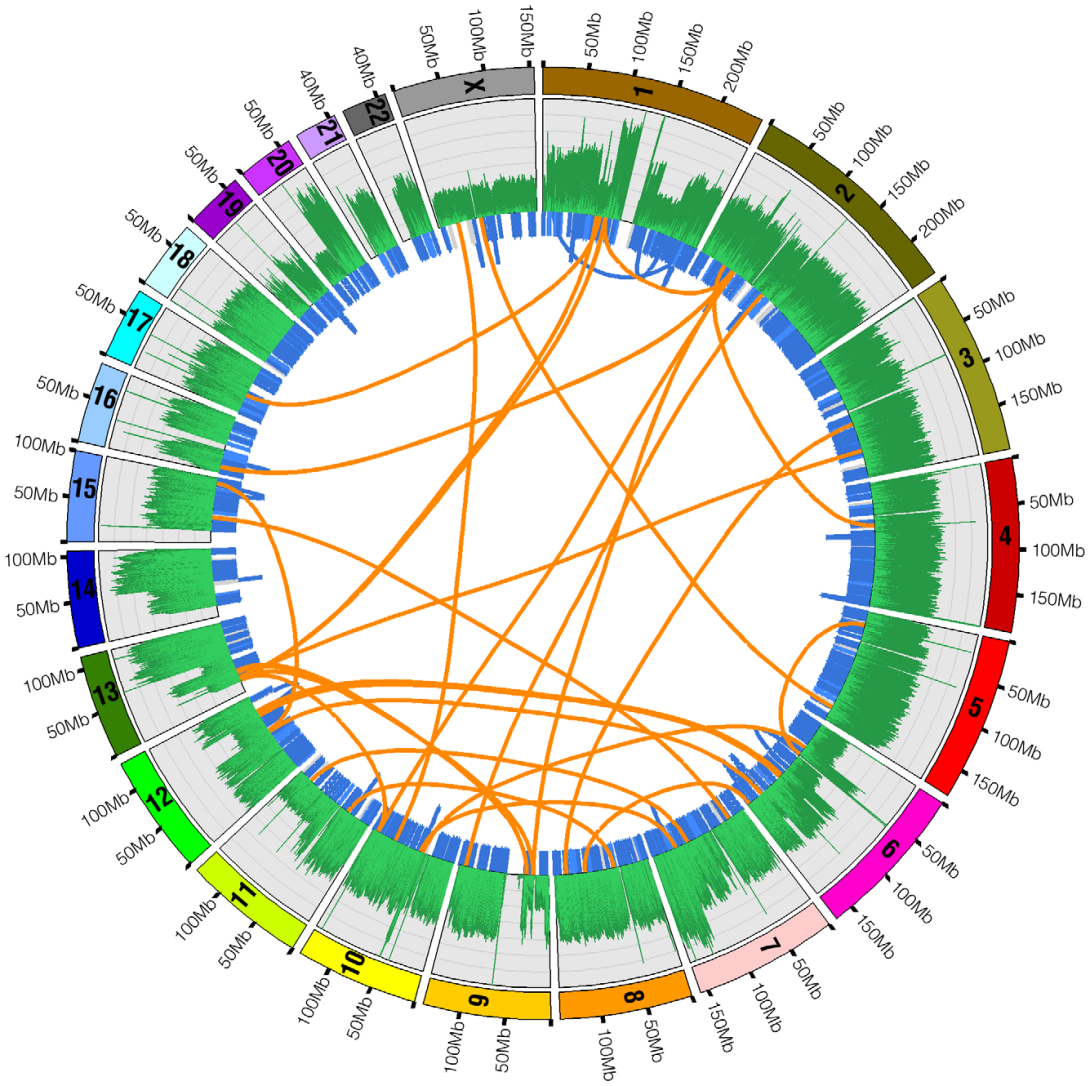
Structural variations in U87MG. Structural variations detected by whole genome sequencing in the U87MG genome are plotted in the Circos program. Orange lines linking two chromosomes represent the 35 interchromosomal translocations. Blue lines around the edge of the circle represent microdeletions and intrachromosomal translocations. The outermost histogram represents sequence coverage and demonstrates how the boundaries of changes in coverage typically coincide with a significant structural variation.

We utilized the predictable insert distance of mate-paired sequence fragments to directly observe structural variations in U87MG. Our target insert size of 1.5kb gave us a normal distribution of paired end insert lengths ranging from 1kb to 2kb with median around 1.25kb and mean around 1.45kb in the actual sequence data (Figure S2). We identified 1,314 large structural variations, including 35 interchromosomal events, 599 complete homozygous deletions (including a large region on chromosome 9 containing CDKN2A/B, which commonly experience homozygous deletions in brain cancer), 361 heterozygous deletion events, and 319 other intrachromosomal events (Table 5). The 599 complete microdeletions summed up to approximately 5.76Mb of total sequence, while the 361 heterozygous microdeletions summed to 5.36Mb of total sequence. Most of the microdeletions were under 2kb in total size. Because of the high sequence coverage and mate pair strategy each event was supported by an average of 138 mate pair reads. Mispairing of the mate pairs did occur occasionally due to molecular chimerism in the library fabrication process, but such reads occur at a low frequency (<1/40 of the reads). Thus, the true rearrangement/deletion events were highly distinct from noise in well-mapped sequences. Interchromosomal events included translocations and large insertion/deletion events where one part of a chromosome was inserted into a different chromosome, sometimes replacing a segment of DNA. All together, these structural variations show a highly complex rearrangement of genomic material in this cancer cell line (Figure 5). All identified structural variants are summarized in Table S2. We note as well that even when breakpoints are within genome-wide common repeats there can be sufficient mapping information to reliably identify the translocation breakpoint (Figure S3).

**Table 5:** Structural variations detected.

Type	# of events	# that span genes (%)	# of affected genes (%)
**Complete deletion**	599	95 (15.9%)	145
**Heterozygous deletion**	361	58 (16.0%)	91
**Interchromosomal translocation**	35	32 (91.4%)	35
**Other intrachromosomal events**	319	146 (45.8%)	166

**Figure 6:**
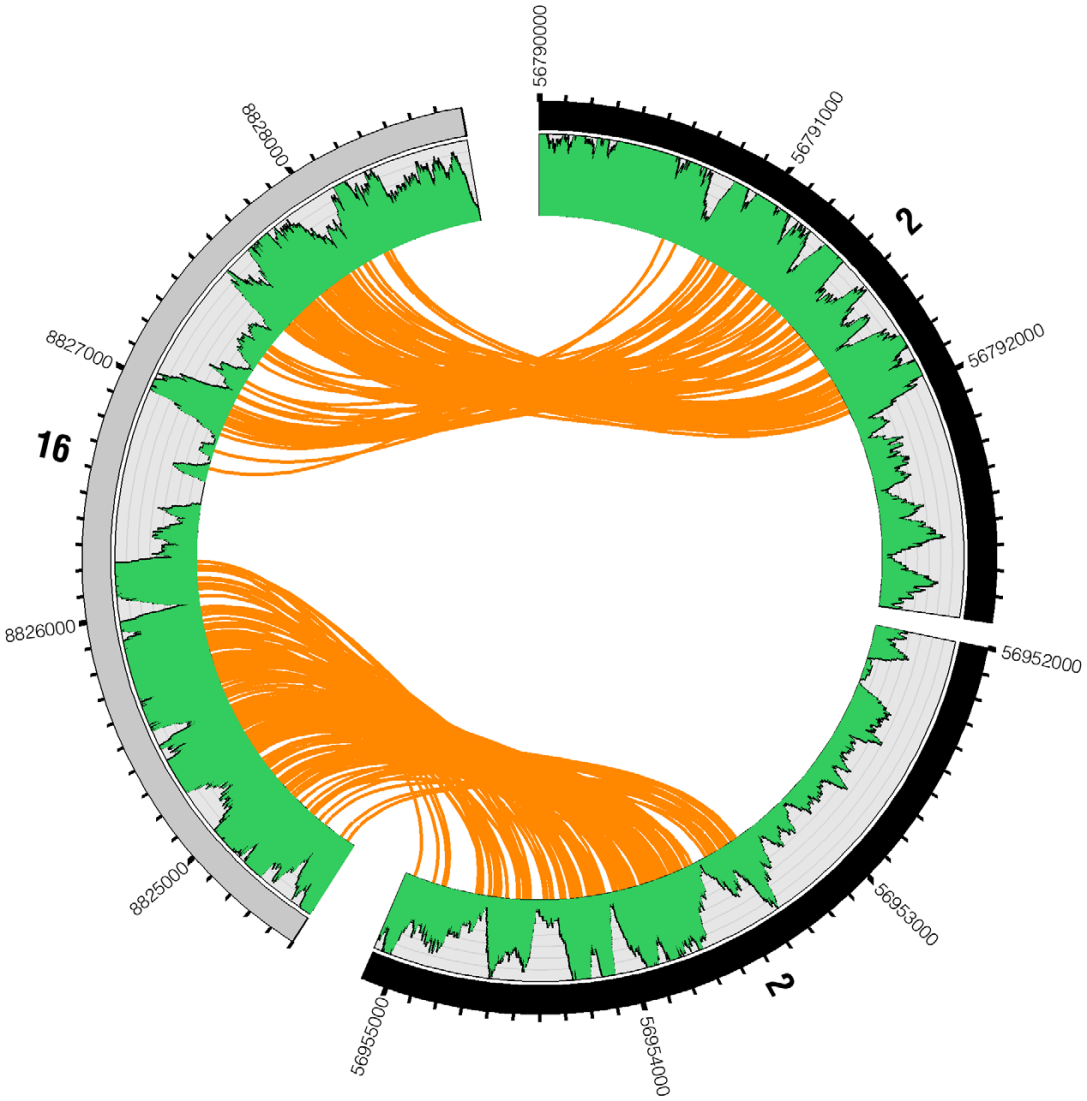
Reads spanning interchromosomal translocation breakpoints. Two genomic breakpoint events are highlighted between chromosomes 2 and 16. The outer ring represents the chromosomes displaying tick marks every 100 bases. The green plot shows base-coverage for each position. Each orange line represents a single mate-pair as a link between one end of a read and its mate-pair. Between the breakpoints on each chromosome (chr2:56792000–56953300 and chr16:8826200–8826700), base coverage drops to about half of what it is on the other side of the event, from two to one copy. This suggests an interchromosomal translocation between chromosomes 2 and 16 resulting in a loss of the genomic material between the translocation breakpoints.

The thirty-five interchromosomal events often coincided with positions of copy number change based on the average base coverage (Figure 5). Figure 6 shows two interchromosomal events between chromosomes 2 and 16. The events on chromosome 16 are less than 1kb apart while those on chromosome 2 are about 160kb apart. Based on the average base coverage, there appears to be a loss of genomic material between the event boundaries on their respective chromosomes, shifting from two to one copy. Although we are unable to determine the origin of such an event, it appears that there was an interchromosomal translocation between chromosomes 2 and 16 with a loss of the DNA between the identified regions on each chromosome.

A subset of 3 translocations were confirmed by amplifying DNA from the breakpoint-spanning region by polymerase chain reaction and sequencing by dideoxy Sanger sequencing (Table S1). Each confirmed the predicted breakpoint to within 100 nucleotides of the correct position. In a subset of cases, unmapped short read fragments could be identified from the shotgun short read data that span the breakpoint and are concordant at base resolution with Sanger sequencing of PCR amplified product spanning the breakpoint

### Genes Affected by Mutations in Coding Sequence

The SNVs and indels identified in U87MG were assessed for their potential to affect protein-coding sequence. We considered variants predicted to be homozygous and to affect the coding sequence of a gene through a frameshift, early termination, intron splice site, or start/stop codon loss mutation as causing a complete loss of that protein. We chose to focus on homozygous null mutations for two major reasons. First, this is an interesting set of genes that we can predict from the whole genome data are non-functional within this commonly used cell line. Although heterozygous mutations can certainly affect gene products in multiple ways, it is difficult to assess their effect from genomic data alone. Second, by cross-referencing such null mutations with known regions of common mutation in gliomas we can pick out specific candidates that are of interest to the glioma community.

Of the 2,384,470 SNVs and 191,743 small indels in U87MG, a total of 332 genes are predicted to have loss-of-function, homozygous mutations as a consequence of small variants (Table S3). Of these, 225 genes contained variants matching alleles annotated in dbSNP (version 129), while 107 contained novel variants not observed in dbSNP.

We further divided these homozygous mutant genes by variant type. Of genes mutated by SNVs, 146 contained variants present in dbSNP while only 8 were knocked out by variants not in dbSNP. The ratio of known SNPs causing loss-of-function mutations to total known SNPs (146/2,140,848 = 6.82×10^−5^) was not significantly different from the ratio of novel SNVs causing loss-of-function mutations over total novel SNVs (8/243,622 = 3.28×10^−5^; p = 0.04). This indicates that many of the possible de novo point mutations may indeed be rare inherited variants made homozygous by chromosomal loss of the normal allele.

In contrast to the trend in SNVs, small indels that homozygously mutated genes were more often novel. There were 79 genes predicted homozygously mutated by indel variants reported in dbSNP while 99 were predicted mutated by novel indels. Despite this trend, however, there was not a significant enrichment of deleterious indels among the novel indels (99/191,743 = 5.16×10^−4^) compared to the known indels (79/116,964 = 6.75×10^−4^; p = 0.08) This suggests that the difference in ratios of novel versus documented SNVs (8 vs. 146) and indels (99 vs. 79) is the result of compositional bias in dbSNP129, which contains a far greater number of SNPs compared to indels.

We also assessed the structural variants in U87MG for whether or not they were likely to affect a gene. Two different criteria were used to determine if translocations and microdeletions impacted a coding region, both predicted to produce an aberrant or nonfunctional protein. Using the UCSC known gene database, we identified 35 genes affected by interchromosomal translocations, 145 affected by complete deletions, 91 affected by heterozygous deletions and 166 affected by other intrachromosomal translocations (Table 4).

Interchromosomal translocation events were significantly enriched for occurring at positions where they would affect genes with 32 out of 35 events (91.4%) occurring within 1kb of a gene (p<0.0001), while only 44.1% of the reference genome is within 1kb of a known gene. In total, intrachromosomal events did not display this enrichment with 145/319 (45.5%) falling within 1kb of a gene (p = 0.67). However, we ran a set of simulations to assess whether microdeletions were enriched to overlap exons because we noted that 585 of our 599 complete microdeletions were less than 10kb in length with a mean size of 1.8kb. We ran 100,000 simulations randomly placing 600 microdeletions of 2kb lengths and determined how many times a microdeletion spanned an exon. In this way, we demonstrated that complete (homozygous) microdeletions under 10kb in size spanned exons slightly more often than by chance with a simulated p-value of .046. Similar assessment of microdeletions greater than 10kb in size did not find evidence of enrichment. These findings suggest that small microdeletions may preferentially occur within genes as opposed to being randomly distributed across the genome, but the signal is not strong from the available data. Genes affected by structural variations are summarized in Table S4.

### Annotation of Relevant Mutated Genes

The annotation tool DAVID was used to further examine the biological significance of the list of likely knockout mutations (including genes affected by SNVs, indels, microdeletions and translocation events) using the EASE analysis module. After gene ontology (GO) analysis, 18 GO terms were nominally enriched and associated with the mutated gene with a p-value < = 0.01 (Table S5). These GO enrichments include cell adhesion (GO:0007155 and GO:0022610), membrane (GO:0044425), and protein kinase regulator activity (GO:0019887).

The list of genes was also compared to the list of cancer-associated genes maintained by the Cancer Gene Census project (http://www.sanger.ac.uk/genetics/CGP/Census/). For SNVs and small indels, eight were observed in the census list, but this is not unexpected given the large number of mutations found in this cell line (p = 0.21). Two CGC genes were affected by complete microdeletions (CDKN2A and MLLT3), and one gene each was affected by heterozygous microdeletions (IL21R) and interchromosomal translocations (SET). These included genes previously annotated as mutated in instances of T cell prolymphocytic leukemia (TCRA and MLLT3), glioma (PTEN), endometrial cancer (PTEN), anaplastic large-cell lymphoma (CLTCL1), prostate cancer (ETV1 & PTEN), Ewing sarcoma (FLI1 and ETV1), desmoplastic small round cell tumor (FLI1), acute lymphocytic leukemia (FLI1 and MLLT3), clear cell sarcoma (FLI1), sarcoma (FLI1), myoepithelioma (FLI1), follicular thyroid cancer (PAX8), non-Hodgkin lymphoma (IL21R), acute myelogenous leukemia (SET), fibromyxoid sarcoma (CREB3L2), melanoma (XPC), and multiple other tumor types (PTEN and CDKN2A).

We also explored the overlap of genes with mutations in GBMs according to the Cancer Genome Atlas (TCGA) with those we predicted are homozygously loss-of-function mutated in U87MG (Table S5). Seven genes mutated in U87MG by SNVs or indels were also found mutated within the TCGA sample (PTEN, LTF, KCNJ16, ABCA13, FLI1, MLL4, DSP). This overlap is not statistically significant (p = 0.16). Ten additional genes overlapped, including two genes mutated by interchromosomal translocations (CNTFR, ELAVL2), three genes mutated by intrachromosomal translocations (ANXA8, LRRC4C, ALDH1A3), and five by homozygous microdeletions (CDKN2A, CDKN2C, MTAP, IFNA21, TMBIM4).

Finally, in order to place the homozygous mutations of U87MG in context relative to GBM mutational patterns as a whole, the Genomic Identification of Significant Targets in Cancer (GISTIC) method [28] was applied to 293 glioblastoma samples with genome wide copy number information available from the TCGA. This yielded a list of significant, commonly deleted regions present across glioblastomas as a group and highlights genes commonly mutated in GBMs. These data indicate that all or parts of chromosomes 1, 6, 9, 10, 13, 14, 15, and 22 are commonly deleted within GBMs as a group. In total, these regions comprise 915,306,764 bases, covering roughly 30 percent of the genome. In order to highlight genes homozygously mutated in U87MG that are within the regions of common loss, we cross-referenced these lists and found that 62/332 (19%) are within the GISTIC defined regions. This does not suggest a significant overlap of homozygously mutated genes in U87MG with commonly deleted regions, but those mutated genes that do overlap may be of increased relevance to cancer. Two of the 62 genes are also in the Cancer Gene Census: PTEN and TCRA. We propose that a subset of the genes mutated in U87 within these commonly deleted regions may be the specific targets of mutation and should be assessed on larger sample sets. (Table S5 and Figure S4).

## Discussion

Reported individual human genome sequencing projects using massively parallel shotgun sequencing with alignment to the human reference genome clearly indicate the practicality of individual whole genome sequencing. However, the monetary cost of data generation, data analysis issues, and the time it takes to perform the experiments have remained substantial limitations to general application in many laboratories. Here we demonstrate enormous improvements in the throughput of data generation. Using a mate-pair strategy and only ten micrograms of input genomic DNA, we generated sufficient numbers of short sequence reads in approximately 5 weeks of machine operation with a total reagent cost of under $30,000. We believe this makes U87MG the least expensive published genome sequenced to date signaling that routine generation of whole genomes is feasible in individual laboratories. Further, the two-base encoding strategy employed within the ABI SOLiD system is a powerful approach for comprehensive analysis of genome sequences and, in concert with BFAST alignment software, is able to identify SNVs, indels, structural variants, and translocations.

Of particular interest in whole-genome resequencing studies such as this one is how much raw data must be produced to sequence both alleles using a shotgun strategy. Here, 107.5Gb of raw data was generated. Of this, 55.51Gb was mapped to unique positions in the reference genome. In effect, this results in a mean base coverage of 10.85× per allele within non-repetitive regions of the genome. Repetitive regions are of course undermapped, as their unique locations are more difficult to determine. This level of oversampling is adequate for high stringency variant calling (error rate less than 5×10^−6^) at 93.71% of heterozygous SNP positions. There may be some biases in library generation resulting in bases that are not successfully covered even if they are relatively unique, but solutions to this may be found in performing multiple sequencing runs with varied library designs, as suggested in other studies [17].

With rapid advances in the generation of massively parallel shotgun short reads, one of the major computational problems faced is the rapid and sensitive alignment of greater than 1 billion paired end reads needed to resequence an individual genome. We demonstrate a practical solution using BFAST, which was able to perform fully gapped local alignment on the two-base encoded data to maximize variant calling in less than 4 days on a 20-node 8-core computer cluster.

Comparing U87MG SNVs with the James Watson [12] and YanHuang [14] genome projects' SNVs displays differences in SNV detection between the three projects. Being derived from a Caucasian individual, U87MG and James Watson are expected to share more SNVs than U87MG and YanHuang. However, when we compared SNVs between U87MG and these two genomes, more SNVs were actually shared between U87MG and YanHuang. Meanwhile, the YanHuang project called significantly more SNVs in total than both our U87MG sequencing project and the James Watson project. These results stress that utilizing different sequencing platforms (U87MG-ABI SOLiD, James Watson-Roche 454, YanHuang-Illumina Solexa), alignment tools (U87MG-BFAST, James Watson-BLAT, YanHuang-SOAP) and analytical approaches results in finding different quantities of SNVs. The higher genomic coverage in our U87MG sequence relative to James Watson and the increased sensitivity of BFAST relative to BLAT and SOAP were counted on to find highly robust variants. This is particularly important when sequencing a cancer genome because of the interest in finding novel cancer mutations as opposed to common polymorphisms.

The genomic sequence demonstrates global differences in variant type across the coding and non-coding portions of the human genome. By increasing the sensitivity of indel detection, we revealed that small indels have mutated genes at a higher rate than SNVs. A larger proportion of the indels identified are predicted to cause a protein coding change compared to SNVs (178/191,743 indels vs. 154/2,384,470 SNVs).

In U87MG, there is a relative increase in 4-base indels genome-wide, which has been observed in other normal genomes [23]–[25] (Figure 2, red bars). However, indels found in coding regions exhibit a bias toward events that are multiples of 3-bases in length (Figure 2, blue bars) presumably selected to maintain reading frame. Thus, many of these events are likely to be polymorphisms and not disease related genomic mutations [25]. Similarly, the nucleotide substitution frequencies demonstrate a bias in coding regions compared to non-coding. Two-thirds of the substitutions were transitions genome-wide, as expected [27], but there was an enrichment of CG->TA transitions in coding regions (Figure 3). It is well established that the most common source of point mutations and SNPs in primates is deamination of methyl-cytosine (meC), causing transition to a thymine (T) [16],[29], and there is circumstantial evidence of that in U87MG's genome as well.

**Figure 7:**
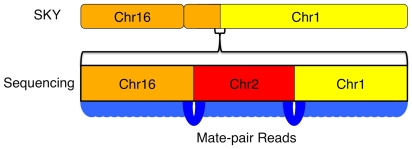
Increased resolution of structural variations by sequencing. Resolution of karyotyping and SKY approaches is not high enough to see the complex nature of this translocation event between chr1 and chr16. With high-resolution whole-genome sequencing, the true structure of the translocation is revealed as mutual translocations between a small fragment of chr2 with chr1 and chr16 on either end.

The resolution of genome-wide chromosomal rearrangements is substantially improved by the mate-pair strategy, coupled with sensitive and independent alignment of the short 50-base reads (Figure 5). Based on published SKY data, we anticipated 7 interchromosomal breakpoints [6]. However, whole-genome mate-paired sequence data revealed the precise chromosomal joins of 35 interchromosomal events, which account for previously observed chromosomal abnormalities in U87MG but at additional finer scale resolution (Figure 5, Figure 6, Figure 7). The translocation events were enriched in genic regions with 32/35 (91.4%) occurring within 1kb of genes. A weaker, but still noticeable enrichment over genes occurs with microdeletions as well, which are generally missed by other experimental techniques like DNA microarrays. Thus, within the overall mutational landscape of this cancer cell line, translocations and structural variants preferentially occurred over genes, supporting a model where cancer mutations occur via structural instability rather than novel point mutations.

Delving into the functional effects of the mutations in U87MG through gene ontology and cross-referencing the literature, we found a large number of known and predicted cancer mutations present in the cell line. There is always a concern when dealing with a cancer cell line that mutations will be more related to its status as a cell line than to the cancer it was derived from. While this remains a concern, the large number of predicted and known cancer genes present in U87MG suggests other genes mutated in it have relevance to cancer as well. Using GISTIC to find regions with common deletions in glioma samples, we highlight 60 genes that are mutated in U87MG and are located in regions that are commonly deleted in GBMs that are not included within the Cancer Gene Census list as potential candidate mutational targets in GBMs (Table S5).

Cancer cell lines are commonly used as laboratory resources to study basic molecular and cellular biology. It is clearly preferable to have complete genomic sequence for these valuable resources. U87MG is the most commonly studied brain cancer cell line and is highly cytogenetically aberrant. While this made the sequencing and mutational analysis more challenging, it serves as a model for future cultured cell line genomic sequencing. Through custom analyses, we found that the mutational landscape of the U87MG genome is vastly more complicated than we would have expected based on the variants discovered in previously published genomes. It is our hope that the increased genomic resolution presented here will direct researchers and clinicians in their work with this brain cancer cell line to create more effective experiments and lead to a greater ability to draw meaningful conclusions in the future.

## Materials and Methods

### Data Sources

The NCBI reference genome (build 36.1, hg18, March 2006), genome annotations, and dbSNP version 129 were downloaded from the UCSC genome database located at http://genome.ucsc.edu. A local mirror of the UCSC genome database (hg18) was used for the subsequent analysis of variants using included gene models and annotations. The Watson genome variants were downloaded from Cold Spring Harbor Laboratory (http://jimwatsonsequence.cshl.edu) with bulk data files available from ftp://jimwatsonsequence.cshl.edu/jimwatsonsequence/. The YanHuang variants were downloaded from the Beijing Genomics Institute at Shenzhen (http://yh.genomics.org.cn/) with bulk data files available from http://yh.genomics.org.cn/download.jsp.

### Sample Preparation

U87MG cells were ordered from ATCC (HTB-14) and cultured in a standard way. Genomic DNA was isolated from cultured U87MG cells using Qiagen Gentra Puregene reagents. DNA was stored at −20C until library generation.

### ABI Sequencing

Long-Mate-Paired Library Construction: The U87MG genomic DNA 2× 50bp long mate-paired library construction was carried out using the reagents and protocol provided by Applied Biosystems (SOLiD 3 System Library Preparation Guide). A similar protocol was reported previously [17]. Briefly, 45ug of genomic DNA was fragmented by HydroShear (Digilab Genomic Solutions Inc) to 1.0–2.5kb. The fragmented DNA was repaired by the End-It DNA End-Repair Kit (Epicentre). Subsequently, the LMP CAP adaptor was ligated to the ends. DNA Fragments between 1.2–1.7kb were selected by 1.0% agarose gel to avoid concatamers and circularized with a biotinylated internal adaptor. Non-circularized DNA fragments were eliminated by Plasmid-Safe ATP-Dependent DNase (Epicentre) and 3ug of circularized DNA was recovered after purification. Original DNA nicks at the LMP CAP oligo/genomic insert border were translated into the target genomic DNA about 100bp by nick translation using E. coli DNA polymerase I. Fragments containing the target genomic DNA and adaptors were cleaved from the circularized DNA by single-strand specific S1 nuclease. P1 and P2 adaptors were ligated to the fragments and the ligated mixture was used to create two separate libraries with 10 cycles of PCR amplification. Finally, 250–300bp fragments were selected to generate mate paired sequencing libraries with average target genomic DNA on each end around 90bp by excision from PAGE gel and use as emulsion PCR template. Templated Beads Preparation: The templated beads preparation was performed using the reagents and protocol from the manufacturer (Applied Biosystems SOLiD 3 Templated Beads Preparation Guide). SOLiD 3 Sequencing: The 2×50b mate-paired sequencing was performed exactly according to the Applied Biosystems SOLiD 3 System Instrument Operation Guide and using the reagents from Applied Biosystems.

### Exon Pull-Down Capture Sequencing with Illumina GAII

We used an array pull-down capture strategy established in our lab [30]. An Agilent custom array for capturing 5,253 “cancer-related” genes was designed through Agilent e-array system (www.agilent.com). Only the amino acid encoding regions were targeted with 60mer oligos spaced center-to-center 20–30bp. The probes were randomly distributed across two separate 244K arrays. The library for cancer gene capture sequencing was generated following the standard Illumina paired-end library preparation protocol. 5ug of genomic DNA was used for the starting material and 250–300bp fragments were size-selected during the gel-extraction step. In the last step, 18 cycles of PCR were performed in multiple tubes to yield 4ug of product and mixed with 50ug of Human Cot-1 DNA (Invitrogen), 52ul of Agilent 10× Blocking Agent, 260ul of Agilent 2× Hybridization Buffer and 10× molar concentration of unpurified Illumina paired-end primer pairs custom made according to the sequences provided by Illumina (Oligonucleotide sequences, 2008, Illumina, Inc: available on request from Illumina). The mix was then diluted with elution buffer for the final volume of 520ul and then incubated at 95°C for 3 min and 37°C for 30min. 490ul of the hybridization mix was added to the array and hybridized in the Agilent hybridization oven (Robins Scientific) for 65 hrs at 65°C, 20rpm. After hybridization, the array was washed according to the Agilent wash procedure A protocol. The second wash was extended to 5 minutes to increase the wash stringency. After washing, the array was stripped by incubating it in the Agilent hybridization oven at 95°C for 10min, 20rpm with 1.09× Titanium Taq PCR Buffer (Clonetech). After the incubation and collection of the solution, 4 tubes of PCR were performed with each tube containing 96ul of the collected solution, 1ul of dNTPs (10mM each), 1ul of Titanium Taq (Clonetech) and Solexa primers, 1ul each. 15 cycles of PCR was performed at the following condition: 30sec at 95°C, (10 sec at 95°C, 30 sec at 65°C, 30 sec at 72°C)×18 cycles, 5 min at 72°C and hold at 4°C. The amplified product was purified using QIAquick PCR Purification Kit and eluted in 30ul of EB. After confirming the size of the amplicon on 2% agarose gel and measuring the concentration, the amplicon was diluted to 10nM, the working concentration for cluster generation. The Illumina flowcell was prepared according to the manufacturer's protocol and the Genome Analyzer was run using standard manufacturer's recommended protocols. The image data produced were converted to intensity files and were processed through the Firecrest and Bustard algorithms (1.3.2) provided by Illumina to call the individual sequence reads.

### ABI SOLiD Sequence Alignment and Consensus Base Calling

We used Blat-like Fast Accurate Search Tool version 0.5.3 (BFAST http://bfast.sourceforge.net) [19] to perform sequence alignment of the two-base encoded reads off the ABI SOLiD to the NCBI human reference genome (build 36.1). Utilizing the local alignment algorithm included in BFAST [31], we were able to simultaneously decode the short reads, while searching for color errors (encoding errors), base changes, insertions, and deletions.

We found candidate alignment locations (CALs) for each end independently. We utilized ten indexes to be robust to up to six color errors, equating to a 12% per-read error rate:

1111111111111111111111

111110100111110011111111111

10111111011001100011111000111111

1111111100101111000001100011111011

111111110001111110011111111

11111011010011000011000110011111111

1111111111110011101111111

111011000011111111001111011111

1110110001011010011100101111101111

111111001000110001011100110001100011111

We also set parameters to use only informative keys when looking up reads in each index (BFAST parameter -K 8), and to ignore reads with too many CALs aggregated across all indexes (BFAST parameter -M 384). If reads mapped to greater than 384 locations, then they were categorized as ‘unmapped’. We then performed local alignment for each of the returned CALs, simultaneously decoding the read from color space searching for color errors (encoding errors), base changes, insertions, and deletions [31]. We choose the “best scoring” alignment, accepting an alignment only if it was at least the equivalent edit distance of two color errors away from the next best alignment. This is approximately similar to a ‘mapping quality’ of 20 or better from the MAQ program output, for reference. We removed duplicate reads using the alignment filtering utility found in DNAA (http://dnaa.sourceforge.net). For single-end and mate-paired reads where only one end mapped, we removed duplicates based on reads having identical stat positions. For mate-paired reads, we removed duplicates where both ends had the same start position.

### Illumina Genome Analyzer Sequence Alignment

Illumina generated sequence was aligned to the NCBI human reference genome (build 36.1) using BFAST with the following parameters applied. Each end of the fragment library was mapped independently to identify CALs, utilizing ten indexes to be robust to errors and variants in the short (typically 36bp) reads:

1111111111111111111111

1111101110111010100101011011111

1011110101101001011000011010001111111

10111001101001100100111101010001011111

11111011011101111011111111

111111100101001000101111101110111

11110101110010100010101101010111111

111101101011011001100000101101001011101

1111011010001000110101100101100110100111

1111010010110110101110010110111011

We also set parameters to use only informative keys when looking up reads in each index (BFAST parameter -K 8), and to ignore reads with too many CALs aggregated across all indexes (BFAST parameter -M 1280). We then performed a standard local alignment for each CAL. Reads were declared mapped if a single unique best scoring alignment was identified within the genome. Duplicate reads were filtered out in the same manner as for the ABI SOLiD data.

### Single Nucleotide Variant and Small Insertion and Deletion Detection

To find SNVs including SNPs and small indels, we assumed the MAQ consensus-calling model [20] utilizing the implementation in SAMtools [21]. We used a value of 0.0000007 for the prior of a difference between two haplotypes (-r parameter). This was chosen based on ROC analysis of a test dataset (data not shown).

### Structural Variation Detection

Structural variations were detected using custom algorithms designed to comprehensively search for groups of mate-pair reads with aberrant paired-end insert size distributions that are consistently identifying a unique structural variant in the genome. We utilized the “dtranslocations” utility in the DNAA package (http://dnaa.sourceforge.net) for the primary structural variation candidate search. The utility first selected all pairs for which each end is uniquely mapped to a single location in the human genome and for which the mate-pair reads are not positioned in the expected size range relative to the consensus genome. Then we filter out false positives that are not consistent with a chromosomal difference on an allele. Briefly, the genome was divided into 500-base bins sequentially stepped 100-bases apart from their start positions. Each bin was then paired with other bins on the basis of containing similar ‘mismapped’ mate-pair reads. The aberrant mate-paired reads were defined as reads that were mapping less than 1000 or greater than 2000 bases apart within the reference genome sequence, which is selected based on the insert size distribution calculated from the aggregate dataset (Figure S2). These were then rank-ordered based on the number of mate-pairs meeting criteria, and the destination bin with the most reads within it was paired with a given source bin to create a ‘binset’. Binsets containing less than 4 reads were filtered out, removing 98.3% of the candidates based on having too little evidence supporting them. The resulting list of filtered binsets was then scanned for clusters of binsets. Binset clusters are groups of binsets where the source bins occur within 2000 bases of each other and the destination bins occur within 2000 bases of each other. Redundant binsets were combined and those binset clusters that contain too few (less than 9 binsets spanning at least 1000 bases) or too many binsets (greater than 29 binsets spanning at most 3000 bases—higher is impossible given our insert size distribution) were removed as artifacts. The resulting binset clusters represent the reads immediately flanking structural breakpoint events. This detection process is currently being automated as Breakway (http://breakway.sourceforge.net), but was done using custom scripts at the time of analysis.

The structural variations were then separated into interchromosomal and intrachromosomal events. Intrachromosomal events of less than 1Mb are assessed for deletion status by averaging base coverage within the bounds of the event and comparing it to base coverage 200kb outside the event on both sides. Those that have average interior base coverage less than 25% of the average exterior base coverage are classified as “complete” deletions. Those with average interior base coverage between 25% and 75% that of average exterior base coverage are classified as “heterozygous deletions” (deletions of at least one copy of the region, but with at least one copy remaining).

### Genes Affected by Mutations in Coding Sequence

Variant calls from the SAMtools pileup tool were first loaded into a SeqWare QueryEngine database and subsequently filtered to produce BED files. This filtering criteria required that a variant be seen at least 4 times and at most 60 times with an observation occurring on each strand at least once. For SNVs we further enforced the criteria that SNVs should only be called in reads lacking indels and the last 5 bases of the reads were also ignored. This reduced the likelihood that spurious mismappings were used to predict SNVs and eliminated the lowest quality bases from consideration. For small indels (<21bp) we enforced a slightly different filter by requiring that any reads supporting an indel were only allowed to contain one contiguous indel and these reads were not considered if the indel occurred on either the beginning or end of the read. These criteria, like the SNV criteria, were used to reduce the likelihood of using mismapped reads or locally misaligned reads in the variant calling algorithm. The elimination of reads with indels at the beginning or end of the read was intended to remove potential alignment artifacts caused by ambiguous gap introduction due to lack of information at the ends to guide proper alignment. Together, these filtering criteria reduced the likelihood that sequencing errors were identified as SNV or indel variants. We used scripts available in the BFAST toolset and SeqWare Pipeline to filter and annotate the variant calls. Variants passing these filters were further annotated by their overlap with dbSNP version 129. Variants were required to share the same genomic position as a dbSNP entry along with matching the allele present in the database to be considered overlapping. Mapping to dbSNP allowed us to filter out known SNPs from de novo variants.

Filtered SNV and indel variants were then analyzed for their affect within the genome that is annotated with gene models. This analysis used scripts from the SeqWare Pipeline project and gene models downloaded from the UCSC hg18 human genome annotation database. Six different gene model sets from hg18 were considered: UCSC genes (knownGene), RefSeq genes (refGene, http://www.ncbi.nlm.nih.gov/RefSeq), Consensus Coding Sequence genes (ccdsGene, http://www.ncbi.nlm.nih.gov/CCDS), Mammalian Gene Collection genes (mgcGenes, http://mgc.nci.nih.gov), Vertebrate Genome Annotation genes (vegaGene, http://vega.sanger.ac.uk), and Ensembl genes (ensGene, http://www.ensembl.org). Each variant was evaluated for overlap with genes from each of the 6 gene models. If overlap was detected the variant was examined and tagged with one or more of the following terms depending on the nature of the event: “utr-mutation”, “coding-nonsynonymous”, “coding-synonymous”, “abnormal-ref-gene-model-lacking-stop-codon”, “abnormal-ref-gene-model-lacking-start-codon”, “frameshift”, “early-termination”, “inframe-indel”, “intron-splice-site-mutation”, “stop-codon-loss”, and/or “start-codon-loss”. The variant was also tagged with the gene symbol and other accessions to facilitate lookups. This information was loaded into a SeqWare QueryEngine database to allow for querying and filtering of the variants as needed.

Genes affected by structural variations were assessed in two ways depending on the structural variation type. For interchromosomal translocation events, a gene was considered “affected” when either end of an interchromosomal translocation event fell in a genic region (including the entire coding region plus 1kb up- or down-stream of the gene's coding region). The same criteria were used for all intrachromosomal translocation events. For events that were classified as complete or heterozygous deletions, a gene was considered affected if all or part of a coding exon was deleted.

### Annotation of Relevant Mutated Genes

Homozygous SNVs, small indels, large deletions, and translocation events for variants that included predicted coding sequence changes were tallied. This became a reference list of variants with serious homozygous mutations that likely completely disrupted, or “knocked out”, the normal function or synthesis of the target protein.

For the SNVs and small indels, a “knockout” variant was defined as a homozygous call by the SAMtools variant caller where the variant was predicted by the SeqWare Pipeline scripts to change coding sequence with one or more of the following annotations: “early-termination”, “frameshift”, “intron-splice-site-mutation”, “start-codon-loss”, and/or “stop-codon-loss”. The “early-termination” event represented a stop codon introduced upstream of the annotated stop codon. The “frameshift” represented an indel that resulted in a shifting of the reading frame of the gene resulting in, typically, early termination and non-sense coding sequence. The “intron-splice-site-mutation” referred to a mutation in the two consensus splice site intronic bases flanking exons (GT at the 5′ splice site and AG at the 3′ splice site). Finally, “stop-codon-loss” and “start-codon-loss” simply refer to variants that interrupt the stop or start codons. We chose to not include “coding-nonsynonymous” and “inframe-indel” annotations in this list of knocked out variants because, while potentially serious as these mutations are, they are not guaranteed to result in an unexpressed or non-functional protein. However, homozygous frameshift, early termination, splice site, and stop/start codon loss mutations are very likely to interrupt a gene's expression and translation to functional protein.

As described above, large microdeletions that removed all or part of an exon and interchromosomal translocation events that fell within 1kb of a gene's coding region were also classified as mutated genes.

Once suspect knockout variants were identified, a mapping process was used to translate one or more variants to the gene symbol. This mapping allowed us to condense multiple variants affecting multiple gene models to a more abbreviated list of gene symbols likely to be affected by these knockout mutations. The mapping from variants to gene symbols used variants identified with gene models from the refGene and the knownGene tables in the UCSC hg18 database and mapped these variants to gene symbols using queries against the name field of the knownGene table and the alias field of the kgAlias table. The UCSC table browser was used to accomplish these queries and map the knownGene identifiers to gene symbols via the kgXref table. A similar approach was used for homozygous large-scale microdeletions and translocation events.

### DAVID/EASE Analysis

The list of knockout genes was uploaded to the Database for Annotation, Visualization, and Integrated Discovery (DAVID, version 2008) to identify enriched Gene Ontology (GO) terms [32]–[33]. Overlap with GO terms from the biological process, cellular component, and molecular function ontologies were considered. The default parameters were used and a p-value cutoff of < = 0.01 was considered significant.

### Cancer Gene Census

The overlap between the Cancer Gene Census genes and those identified as knockouts in U87MG were compared. The Cancer Gene Census project is an ongoing effort to catalog genes with mutations that have been implicated in cancer [34]. It is a highly curated list that includes annotations for each gene including tumor types, class of mutations, and other genetic properties. We used the gene symbol list from the September 30^th^, 2009 complete working list, which includes 412 gene symbols.

### TCGA

The overlap between mutations in the Cancer Genome Atlas (TCGA) and those identified as knockouts in U87MG was analyzed. TCGA is an ongoing effort to understand the molecular basis of cancer through large-scale copy number analysis, expression profiling, genome sequencing, and methylation studies among other techniques [4]. It provides information on mutations found by Sanger sequencing on many patient samples. For glioblastoma this includes sequence data aberrations detected in 158 patient samples and 1,177 genes.

### Genomic Identification of Significant Targets in Cancer

The Genomic Identification of Significant Targets in Cancer (GISTIC) method was used to find significant areas of deletion in 293 samples from the TCGA [24]. The GISTIC technique was designed to identify and analyze chromosomal aberrations across a set of cancer samples, based on the amplitude of the aberrations as well as the frequency across samples. This approach produced a series of commonly deleted regions across the set of TCGA GBMs. To calculate the areas of deletion, we used 293 Affymetrix SNP 6.0 samples segmented using the GLAD SNP analysis module [35]. Default parameters of GISTIC were used. GISTIC produces peak limits, wide peak limits, and in addition broader region limits. These commonly deleted broader regions were then scanned for predicted knockout genes in U87MG.

### Indel Size Distribution and Nucleotide Substitution Frequencies

The distribution of small indel sizes was examined for both deletions and insertions. Indels classified as affecting coding-sequence by the SeqWare Pipeline (see above) were compared to those outside coding regions. Raw counts were collected, recalculated as percents of total, and compared directly.

Similarly, nucleotide substitution frequency was examined for SNVs from U87MG both genome-wide and only in coding regions. Once binned appropriately, the SNV nucleotide substitutions were counted, tallied in a table, and graphed as percents of total.

### Individual Genome Comparison

Variants from the Watson and Yan Huang genome were downloaded from each respective project from the following URLs: ftp://jimwatsonsequence.cshl.edu/jimwatsonsequence/watson-454-snp-v01.txt.gz and http://yh.genomics.org.cn/do.downServlet?file=data/snps/yhsnp_add.gff. These files contained variant calls for each genome along with annotations describing the variant as novel or occurring in dbSNP. The Watson genome only contained SNV calls so our comparison was limited to just SNVs. The Yan Huang genome also contained calls indicating heterozygous or homozygous. However, a variant was considered to match between genomes regardless of zygosity state. We compared the overlap of the U87MG genome, dbSNP and each of these genomes in turn. SNVs from U87MG that were considered for comparison had to meet our criteria; variants had to be observed at least 4 times, at most 60 times, at least once per strand, and with a minimum phred score of 10. SNVs in the three-way comparison were said to match if the position and allele matched between the genomes. If both variants matched between U87MG and the other genome and one was annotated in dbSNP, then the other was considered in dbSNP as well. If neither contained annotations from dbSNP the variant was considered novel. A similar process was carried out for variants distinct to each genome. The results were recorded as Venn diagrams showing the overlap between dbSNP, U87MG, and the Watson or Yan Huang genome.

### Illumina SNP Chip

Genomic DNA from U87MG was submitted to the Southern California Genotyping Consortium to be run on the Illumina Human 1M-Duo BeadChip, which consists of 1,199,187 probes scattered across the human genome. The Illumina Beadstudio program was used to analyze the resulting intensity data. Loss of heterozygosity was determined by analyzing B-allele frequency as determined by the Beadstudio program. Normal two-copy regions of the genome are represented by long stretches of probes with B-allele frequencies of 0, 0.5 or 1. Regions of LOH, on the other hand, deviate from this pattern significantly. Copy number was determined by looking at probe intensity.

### Sanger Sequencing Validation

Primers for validation were designed by targeting regions immediately flanking the event predicted by our whole genome sequence analysis using the Primer3 tool (http://frodo.wi.mit.ed/primer3/). Polymerase chain reaction was performed following standard protocols using Finnzymes Phusion Hot-Start High Fidelity polymerase. Products were run on 2% agarose gel electrophoresis and product purity and size was assessed by staining with ethidium bromide. Sanger sequencing was performed at the UCLA Genotyping and Sequencing core facility using an ABI 3730 Capillary DNA Analyzer. Sequence trace files were analyzed using Geospiza FinchTV. Validation status and PCR primers are listed in Table S1.

### Data Deposition and Availability

Intensities, quality scores, and color space sequence for the genomic sequence of U87 SOLiD were uploaded to the Sequence Read Archive under the accession SRA009912.1/Sequence of U87 Glioblastoma Cell-line. Intensities, quality scores, and nucleotide space sequence for the exon capture U87 Illumina sequence were also uploaded to the Short Read Archive under the same accession. For both datasets, alignment files have been uploaded to the Short Read Archive as additional analysis results.

Variant calls for both datasets are available via a SeqWare QueryEngine web service at http://genome.ucla.edu/U87. This tool allows for querying the variants using a variety of search criteria including coverage, mutational consequence, gene symbol, and others. SeqWare QueryEngine produces results in both BED and WIG format making it compatible with the majority of genome browsers such as the UCSC genome and table browsers. Variant data will be uploaded to SRA as metadata along with the raw sequences. For the whole genome SOLiD alignment, small indels (<21bp), SNVs, large deletions, and translocation events can be queried. For the exon capture Illumina alignment, small indels and SNVs can be queried.

### Software Availability

Most software used for this project is open-source and freely available. We created two software projects that were instrumental in the analysis of the U87MG data: BFAST and SeqWare. The color- and nucleotide-space alignment tool BFAST can be downloaded from http://bfast.sourceforge.net and many of our alignment filtering as well as the primary step in structural variation detection can be found in the DNAA package at http://dnaa.sourceforge.net. The SeqWare software project was used throughout the analysis of variant calls. We used the SeqWare LIMS tool for sample tracking, the SeqWare Pipeline analysis programs for annotating variants with dbSNP status and mutational consequence predictions, and SeqWare QueryEngine was used to database and query variant calls and annotations. This software and documentation can be downloaded from http://seqware.sourceforge.net.

## Supporting Information

Figure S1 Concordance between Solexa capture data and SOLiD whole genome data. The left plot displays the SNP call concordance between each experiment (Solexa capture data in blue, SOLiD whole genome data in red) with the Illumina 1M Beadchip microarray for the 8.5Mb of sequence pulled down in the capture experiment. The right plot displays concordance of the non-reference (mutant) allele calls with the array data for those regions.
(0.43 MB TIF)

Figure S2 Paired end insert size distribution. Empirical paired end insert size distribution for reads where both ends aligned with duplicates removed.
(0.41 MB TIF)

Figure S3 Alignment is robust against genome-wide repeat elements. Circos plot [35] of reads spanning a complete microdeletion on chromosome 2, bases 201855000–201858000, are shown in dark blue, with the normal reads in the surrounding region in light blue. The green plot shows base-coverage at each position. The outermost track shows the structure of a gene, CASP8, overlapping this region (large boxes-exons, lines-introns). The track containing black and red boxes shows genome-wide repeat elements (black-LINE, red-SINE). Note the high density of reads even over conserved LINE elements. Some SINE elements do demonstrate a drop in alignments, but these do not prevent the identification of structural variation-spanning reads.
(0.21 MB TIF)

Figure S4 Commonly deleted regions in GBM according to GISTIC. This deletion plot shows significant regions of deletion in 293 GBM samples from the TCGA. The top of the plot shows the G-score and the bottom shows the q-values. G-score reflects the frequency and amplitude of the deletion. Q-values greater than 0.25 were considered significant. Overlap of genes mutated in U87 via SNVs or Indels and broad regions of deletion are considered to be likely cancer targets. This includes all or part of chromosomes 1, 6, 9, 10, 13, 14, 15, and 22.
(0.43 MB TIF)

Table S1 PCR and dideoxy sequencing validation. A list of the variants that were validated by PCR and dideoxy sequencing including primers used, varient location, and validation status.
(0.03 MB XLS)

Table S2 Structural variants in U87MG. All structural variants listed as regions immediately flanking the genomic breakpoint.
(0.18 MB XLS)

Table S3 Genes knocked out by SNVs/Indels. List of all genes predicted to be knocked out by SNVs and Indels in U87MG.
(0.20 MB XLS)

Table S4 Genes affected by structural variants. List of all genes predicted to be affected by structural variants in U87MG.
(0.48 MB XLS)

Table S5 Annotation of mutated genes. Lists of genes predicted to be mutated in U87MG annotated by various cancer-related gene databases.
(0.17 MB XLS)
