# Correction: U87MG Decoded: The Genomic Sequence of a Cytogenetically Aberrant Human Cancer Cell Line

**DOI:** 10.1371/journal.pgen.1007392

**Published:** 2018-05-16

**Authors:** Michael James Clark, Nils Homer, Brian D. O'Connor, Zugen Chen, Ascia Eskin, Hane Lee, Barry Merriman, Stanley F. Nelson

This Research Article [1] reports the sequencing of “U87MG” cells, which were acquired from ATCC. It has come to the authors’ attention that recent work from Allen et al. reveals that the U87 cell line used in this study is distinct from the original U87MG cell line [2]. Additional analyses by Allen et al. indicate that the cell line presented in the work is of (or likely of) glioblastoma cell origin. The conclusions in the manuscript remain unaltered.

“U87MG” is incorrect in the title, and in all other instances in the text. In all of these cases, the cell line referred to as “U87MG” should be called “U87(ATCC)”. The correct title is: U87(ATCC) Decoded: The Genomic Sequence of a Cytogenetically Aberrant Human Cancer Cell Line. The correct citation is: Clark MJ, Homer N, O'Connor BD, Chen Z, Eskin A, Lee H, et al. (2010) U87(ATCC) Decoded: The Genomic Sequence of a Cytogenetically Aberrant Human Cancer Cell Line. PLoS Genet 6(1): e1000832. doi:10.1371/journal.pgen.1000832.

## References

[1] Clark MJ, Homer N, O'Connor BD, Chen Z, Eskin A, Lee H, et al (2010) U87MG Decoded: The Genomic Sequence of a Cytogenetically Aberrant Human Cancer Cell Line. PLoS Genet 6(1): e1000832 doi: 10.1371/journal.pgen.1000832 20126413 10.1371/journal.pgen.1000832 PMC2813426

[2] Allen M, Bjerke M, Edlund H, Nelander S, Westermark B. (2016) Origin of the U87MG glioma cell line: Good news and bad news. Science translational medicine. 2016;(8): 354: 354re3 doi: 10.1126/scitranslmed.aaf6853 27582061 10.1126/scitranslmed.aaf6853

