# Supplementary figures and images for: U87MG Decoded: The Genomic Sequence of a Cytogenetically Aberrant Human Cancer Cell Line
Source: PLoS Genet. 2010 Jan 29;6(1):e1000832. doi: 10.1371/journal.pgen.1000832 (PMC2813426; doi:10.1371/journal.pgen.1000832)

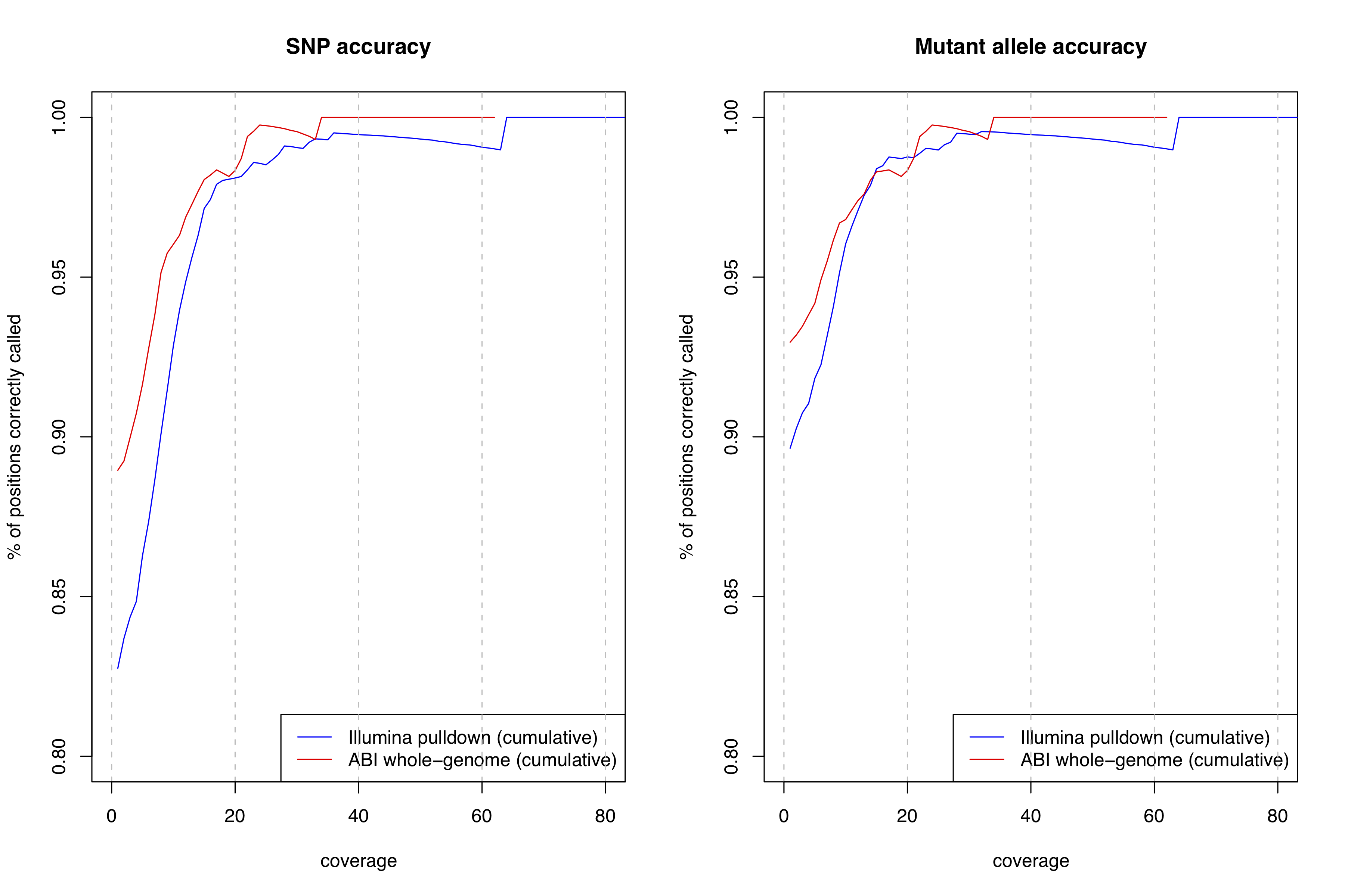

Supplement: Figure S1 — Concordance between Solexa capture data and SOLiD whole genome data. The left plot displays the SNP call concordance between each experiment (Solexa capture data in blue, SOLiD whole genome data in red) with the Illumina 1M Beadchip microarray for the 8.5Mb of sequence pulled down in the capture experiment. The right plot displays concordance of the non-reference (mutant) allele calls with the array data for those regions. (0.43 MB TIF) [file pgen.1000832.s001.tif]

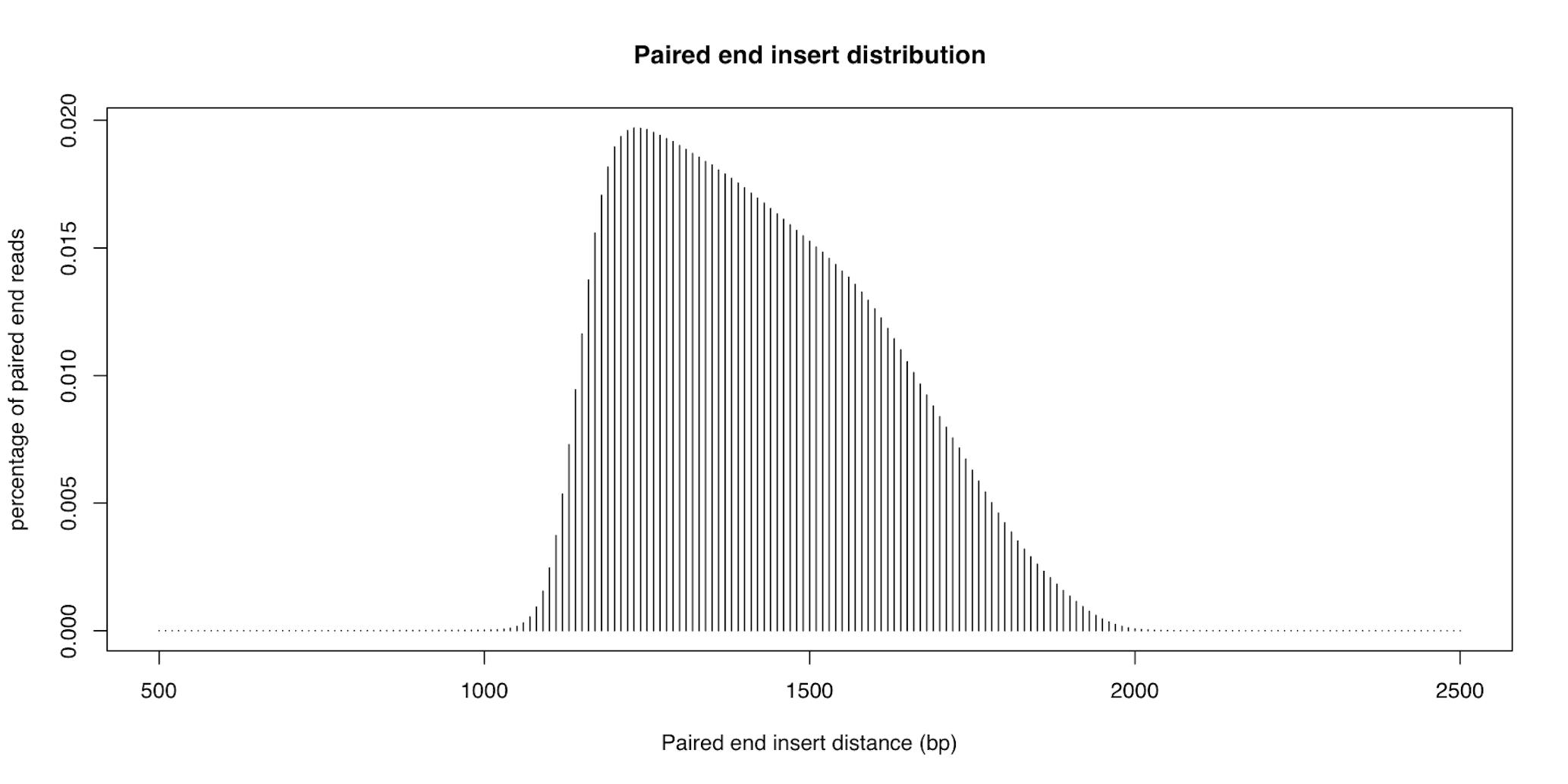

Supplement: Figure S2 — Paired end insert size distribution. Empirical paired end insert size distribution for reads where both ends aligned with duplicates removed. (0.41 MB TIF) [file pgen.1000832.s002.tif]

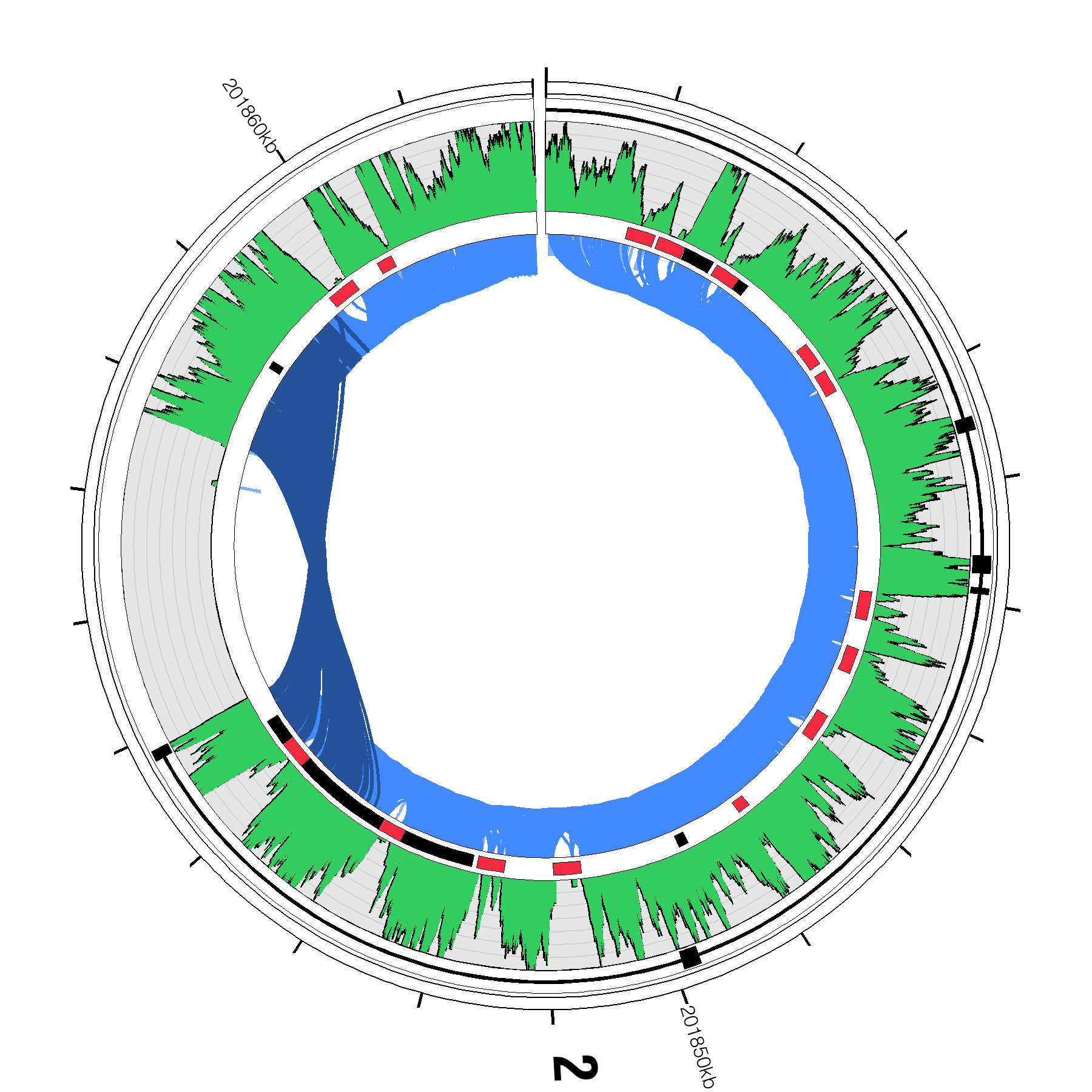

Supplement: Figure S3 — Alignment is robust against genome-wide repeat elements. Circos plot [35] of reads spanning a complete microdeletion on chromosome 2, bases 201855000–201858000, are shown in dark blue, with the normal reads in the surrounding region in light blue. The green plot shows base-coverage at each position. The outermost track shows the structure of a gene, CASP8, overlapping this region (large boxes-exons, lines-introns). The track containing black and red boxes shows genome-wide repeat elements (black-LINE, red-SINE). Note the high density of reads even over conserved LINE elements. Some SINE elements do demonstrate a drop in alignments, but these do not prevent the identification of structural variation-spanning reads. (0.21 MB TIF) [file pgen.1000832.s003.tif]

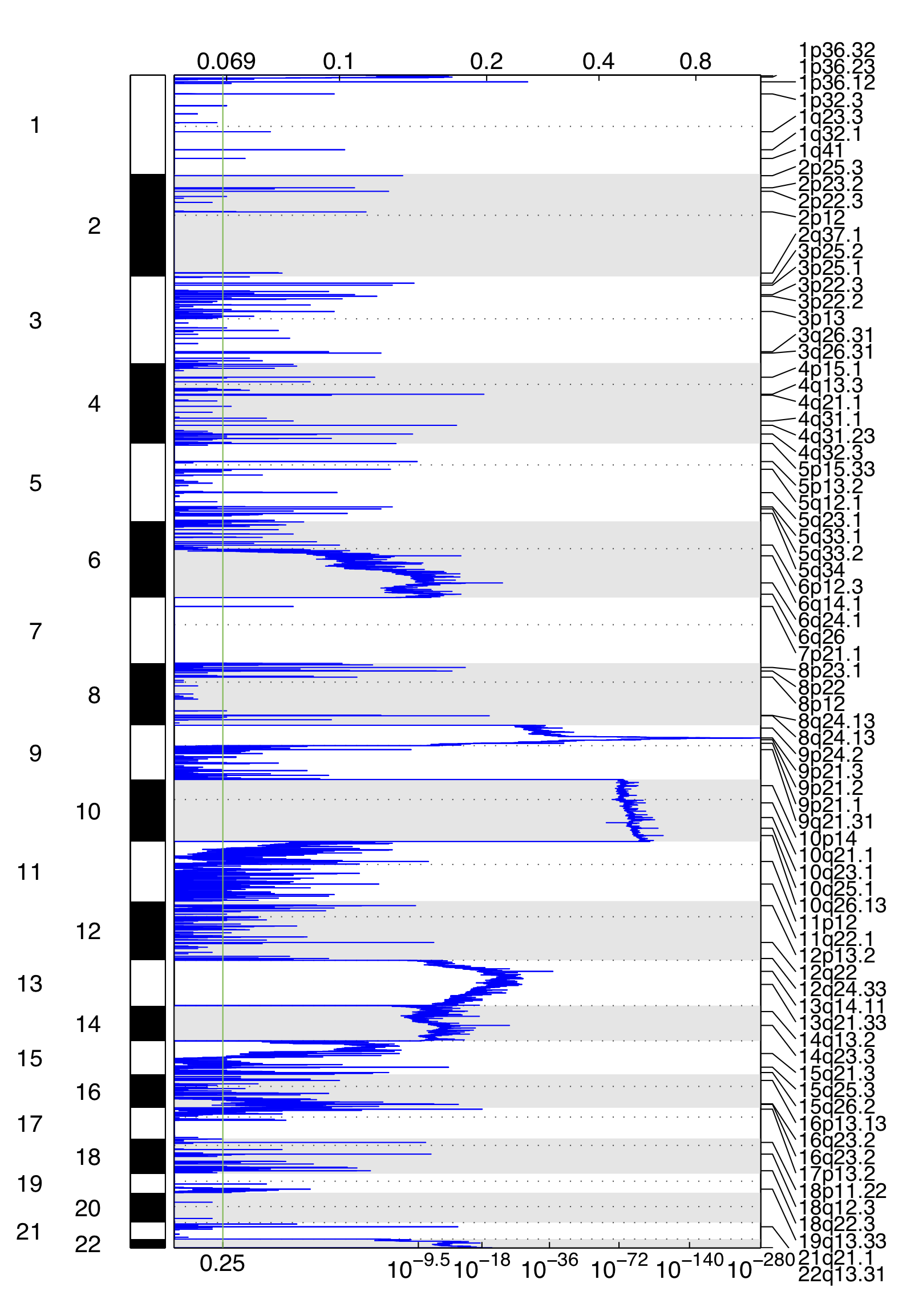

Supplement: Figure S4 — Commonly deleted regions in GBM according to GISTIC. This deletion plot shows significant regions of deletion in 293 GBM samples from the TCGA. The top of the plot shows the G-score and the bottom shows the q-values. G-score reflects the frequency and amplitude of the deletion. Q-values greater than 0.25 were considered significant. Overlap of genes mutated in U87 via SNVs or Indels and broad regions of deletion are considered to be likely cancer targets. This includes all or part of chromosomes 1, 6, 9, 10, 13, 14, 15, and 22. (0.43 MB TIF) [file pgen.1000832.s004.tif]
